# Pembrolizumab or pembrolizumab plus chemotherapy versus standard of care chemotherapy in patients with advanced gastric or gastroesophageal junction adenocarcinoma: Asian subgroup analysis of KEYNOTE-062

**DOI:** 10.1093/jjco/hyac188

**Published:** 2022-12-18

**Authors:** Hironaga Satake, Keun-Wook Lee, Hyun Cheol Chung, Jeeyun Lee, Kensei Yamaguchi, Jen-Shi Chen, Takaki Yoshikawa, Kenji Amagai, Kun-Huei Yeh, Masahiro Goto, Yee Chao, Ka-On Lam, Shi Rong Han, Shinichi Shiratori, Sukrut Shah, Kohei Shitara

**Affiliations:** Department of Medical Oncology, Kobe City Medical Center General Hospital, Kobe City, Japan and Department of Medical Oncology, Kochi Medical School, Kochi, Japan; Department of Internal Medicine, Seoul National University College of Medicine, Seoul National University Bundang Hospital, Seongnam, South Korea; Department of Medical Oncology, Yonsei Cancer Center, Yonsei University College of Medicine, Seoul, South Korea; Division of Hematology/Oncology, Samsung Medical Center Sungkyunkwan University School of Medicine, Seoul, South Korea; Department of Gastroenterological Chemotherapy, The Cancer Institute Hospital of JFCR, Tokyo, Japan; Division of Hematology-Oncology, Chang Gung Memorial Hospital, Tao-Yuan, Taiwan and College of Medicine, Chang Gung University, Tao-Yuan, Taiwan; Department of Gastric Surgery, National Cancer Center Hospital, Tokyo, Japan; Department of Gastroenterology, Ibaraki Prefectural Central Hospital, Ibaraki, Japan; Department of Oncology, National Taiwan University Hospital and Graduate Institute of Oncology, National Taiwan University College of Medicine, Taipei, Taiwan; Cancer Chemotherapy Center, Osaka Medical and Pharmaceutical University Hospital, Osaka, Japan; Department of Oncology, Taipei Veterans General Hospital, Taipei City, Taiwan; Department of Clinical Oncology, LKS Faculty of Medicine, The University of Hong Kong, Queen Mary Hospital, Hong Kong; Department of Medical Oncology, MSD K.K., Tokyo, Japan; Department of Medical Oncology, MSD K.K., Tokyo, Japan; Department of Medical Oncology, Merck & Co., Inc., Rahway, NJ, USA; Department of Gastrointestinal Oncology, National Cancer Center Hospital, Kashiwa, Japan; Department of Immunology, Nagoya University Graduate School of Medicine, Nagoya, Japan

**Keywords:** pembrolizumab, chemotherapy, gastric cancer, gastrooesophageal junction cancer, Asian patients

## Abstract

**Objective:**

First-line pembrolizumab with/without chemotherapy versus chemotherapy was evaluated in programmed death ligand 1 combined positive score ≥1, locally advanced/unresectable or metastatic gastric cancer/gastrooesophageal junction cancer in the KEYNOTE-062 study. We present results for patients enrolled in Asia.

**Methods:**

Eligible patients were randomly assigned 1:1:1 to pembrolizumab 200 mg, pembrolizumab plus chemotherapy (cisplatin + 5-fluorouracil or capecitabine) or placebo plus chemotherapy Q3W. End points included overall survival (primary) in combined positive score ≥1 and combined positive score ≥10 populations and safety and tolerability (secondary).

**Results:**

A total of 187 patients were enrolled in Asia (pembrolizumab, *n* = 62; pembrolizumab plus chemotherapy, *n* = 64; chemotherapy, *n* = 61). Compared with the global population, higher proportions of patients had Eastern Cooperative Oncology Group performance status 0 and a diagnosis of stomach cancer. In the programmed death ligand 1 combined positive score ≥1 population, median overall survival was numerically longer with pembrolizumab versus chemotherapy (22.7 vs 13.8 months; hazard ratio, 0.54; 95% confidence interval, 0.35–0.82) and pembrolizumab plus chemotherapy versus chemotherapy (16.5 vs 13.8 months; hazard ratio, 0.78; 95% confidence interval, 0.53–1.16). In the programmed death ligand 1 combined positive score ≥10 population, median overall survival was also numerically longer with pembrolizumab versus chemotherapy (28.5 vs 14.8 months; hazard ratio, 0.43; 95% confidence interval, 0.21–0.89) and pembrolizumab plus chemotherapy versus chemotherapy (17.5 vs 14.8 months; hazard ratio, 0.86; 95% confidence interval, 0.45–1.64). The grade 3–5 treatment-related adverse event rate was 19.4%, 75.8% and 64.9% for patients receiving pembrolizumab, pembrolizumab plus chemotherapy and chemotherapy, respectively.

**Conclusions:**

This post hoc analysis showed pembrolizumab monotherapy was associated with numerically improved overall survival and a favourable tolerability profile versus chemotherapy in Asians with programmed death ligand 1–positive advanced gastric cancer/gastrooesophageal junction cancer.

This study is registered with ClinicalTrials.gov, NCT02494583.

## Teaser

Subgroup analysis of patients enrolled in Asia in the KEYNOTE-062 study showed first-line pembrolizumab versus chemotherapy improved overall survival and had a favourable tolerability profile in programmed death ligand 1–positive advanced gastric cancer/gastrooesophageal junction cancer.

## Introduction

Gastric cancer (GC) and gastrooesophageal junction cancer (GEJC) is the fifth most frequently diagnosed cancer and the third leading cause of cancer death globally (1,2). GC remains one of the most common cancers in Asia, where it is the third most frequently diagnosed cancer and the second leading cause of cancer death (3). In particular, Japan, China and Korea have the highest incidence of GC in the world (3), highlighting the major healthcare challenge of GC/GEJC in Eastern Asia.

The standard of care for the 85% of patients with unresectable, locally advanced/metastatic GC/GEJC in Asia is doublet or triplet fluoropyrimidine- and platinum-based chemotherapy in the first-line treatment setting (4). Median overall survival (OS) with first-line chemotherapy is ~12 months (5) and is longer in patients in Asia (≥14 months) (6–8). For the approximately 15% of patients with GC expressing human epidermal growth factor receptor 2 (HER2)/Erb-B2 receptor tyrosine kinase 2 (ERBB2) (9,10), the anti-HER2 monoclonal antibody trastuzumab when added to fluoropyrimidine- and platinum-based chemotherapy offers a targeted therapeutic option. However, there is still an unmet need for more effective treatments with lower toxicity for patients diagnosed with advanced GC/GEJC.

The anti–programmed death 1 (PD-1) monoclonal antibody pembrolizumab first demonstrated antitumour activity in previously untreated and treated patients with GC/GEJC in the multicohort phase 2 KEYNOTE-059 study (11,12). Further, investigations in the phase 3 KEYNOTE-061 study suggested that the efficacy of pembrolizumab as a second-line treatment in previously treated patients with GC/GEJC may depend on the degree of expression of tumour programmed death ligand 1 (PD-L1) (13). The efficacy and safety of first-line pembrolizumab (with or without chemotherapy) was subsequently compared with chemotherapy alone in patients with PD-L1–positive (combined positive score [CPS] ≥1) advanced GC/GEJC in the phase 3 KEYNOTE-062 trial (14). In this setting, OS with pembrolizumab monotherapy was noninferior to chemotherapy in patients with a PD-L1 CPS ≥1 and provided durable responses and a survival benefit in patients with PD-L1 CPS ≥10 compared with chemotherapy alone and irrespective of microsatellite instability (MSI) status (14). Pembrolizumab plus chemotherapy was not superior to chemotherapy for OS in either the CPS ≥1 or the CPS ≥10 populations. Furthermore, fewer treatment-related adverse events (AEs) were reported by patients randomly assigned to pembrolizumab monotherapy compared with chemotherapy (14). Patients enrolled in the KEYNOTE-062 trial were stratified by geographic region (Europe/North America/Australia vs Asia vs rest of world), and a prespecified subgroup analysis suggested an enhanced survival benefit with pembrolizumab for patients from Asia compared with patients from other regions (14).

Given the differences in median OS and in regional use of second-line chemotherapy, we conducted a post hoc subgroup analysis to further describe the efficacy and safety of pembrolizumab (with or without chemotherapy) compared with chemotherapy alone in patients with GC/GEJC who were enrolled in Asia in the KEYNOTE-062 study.

## Material and methods

### Trial design, patients and treatment

Full details of the phase 3 KEYNOTE-062 study (ClinicalTrials.gov, NCT02494583) have been published (14). In brief, eligible patients had histologically confirmed, locally advanced/unresectable or metastatic GC/GEJC and no prior (neo)adjuvant therapy ≥6 months before randomization. Patients had to have tumours that were HER2 negative with a PD-L1 CPS ≥1 at randomization. Patients were randomly assigned 1:1:1 to receive pembrolizumab 200 mg every 3 weeks (Q3W), pembrolizumab plus chemotherapy (cisplatin 80 mg/m^2^ on day 1 plus fluorouracil 800 mg/m^2^/day on days 1–5 or capecitabine 1000 mg/m^2^ twice daily on days 1–14 Q3W), or placebo plus chemotherapy. Treatment continued until documented disease progression, unacceptable toxicity or physician/patient withdrawal, or 35 administrations (~2 years) of pembrolizumab or placebo. The current analysis focused on the subgroup of patients enrolled at Asian sites.

All procedures followed were in accordance with the ethical standards of the responsible committee on human experimentation (institutional and national) and with the Helsinki Declaration of 1964 and later versions. The study protocol and all amendments were approved by the appropriate ethics committee at each centre. The study was conducted in accordance with the protocol, its amendments, and standards of Good Clinical Practice. All patients provided written informed consent.

### Assessments and outcomes

Tumour responses were assessed using Response Evaluation Criteria in Solid Tumours, version 1.1 (RECIST v1.1), by blinded independent central review (BICR) every 6 weeks. Adverse events were assessed throughout the study and at 30 days after treatment discontinuation (90 days for serious AEs and immune-mediated AEs and infusion reactions) and were graded according to the National Cancer Institute Common Terminology Criteria for Adverse Events (version 4.0). PD-L1 expression was centrally assessed during screening using PD-L1 IHC 22C3 pharmDx (Agilent) and was scored using CPS (the number of PD-L1–staining cells [tumour cells, lymphocytes, macrophages] divided by the total number of viable tumour cells, multiplied by 100).

The current analysis evaluated OS, progression-free survival (PFS), objective response rate (ORR) and duration of response (DOR) based on BICR assessment per RECIST v1.1 in patients with PD-L1 CPS ≥1 (intention-to-treat population) and in patients with PD-L1 CPS ≥10. Safety and tolerability were also evaluated.

### Statistical analysis

Efficacy was evaluated in all randomly assigned patients in Asia and safety was evaluated in all randomly assigned patients in Asia who received ≥1 dose of study treatment. OS, PFS and DOR were estimated using the nonparametric Kaplan–Meier method and rules for censoring, and between-arm differences in OS and PFS were assessed using a log-rank test. A Cox proportional hazards model with the Efron method of handling ties was used to estimate hazard ratios (HRs) and associated 95% confidence intervals (CIs). Statistical comparisons for efficacy were not performed. The data cut-off date for this analysis was 26 March 2019.

## Results

### Patients

Between 18 September 2015, and 26 May 2017, 763 patients (pembrolizumab, *n* = 256; pembrolizumab plus chemotherapy, *n* = 257; chemotherapy, *n* = 250) were enrolled in KEYNOTE-062; 187 were enrolled at Asian sites (*n* = 62; 64; and 61, respectively) with most patients from Japan (55.1%) and Republic of Korea (26.7%). Baseline characteristics were generally well balanced between treatment arms (Table 1). The median age of all patients enrolled in Asia was 66.0 years (range, 28–85); most patients were male (73.3%) and had adenocarcinoma of the stomach (87.7%). A total of 39.6% of patients had PD-L1 CPS ≥10 tumours. High microsatellite instability (MSI-H) was present in 5.3% of tumours. At the data cut-off date of 26 March 2019, the median study follow-up, defined as the time from randomization to the date of death or database cut-off date, was 16.9 months (range, 0.2–41.0). A total of 52 of 62 patients (83.9%) in the pembrolizumab arm, 43 of 62 (69.4%) in the pembrolizumab plus chemotherapy arm, and 42 of 57 (73.7%) in the chemotherapy arm received subsequent anticancer therapy (Supplementary Table 1).

**Table 1:** Baseline characteristics of patients enrolled in Asia in the KEYNOTE-062 study

Characteristic	Pembrolizumab *n* = 62	Pembrolizumab + Chemotherapy *n* = 64	Chemotherapy *n* = 61
Age, median (range), years	64.5 (28–83)	65.0 (34–83)	67.0 (37–85)
Male, *n* (%)	46 (74.2)	50 (78.1)	41 (67.2)
Region of enrolling site, *n* (%)			
Japan	38 (61.3)	33 (51.6)	32 (52.5)
Korea	17 (27.4)	16 (25.0)	17 (27.9)
Taiwan	6 (9.7)	11 (17.2)	10 (16.4)
Hong Kong	1 (1.6)	4 (6.3)	2 (3.3)
Eastern Cooperative Oncology Group performance status 1, *n* (%)	19 (30.6)	21 (32.8)	24 (39.3)
Gastrectomy, *n* (%)	18 (29.0)	17 (26.6)	17 (27.9)
Metastatic disease, *n* (%)	60 (96.8)	61 (95.3)	60 (98.4)
Tumour size at baseline (above median),^a^ *n* (%)	22 (35.5)	17 (26.6)	21 (34.4)
Programmed death ligand 1 (PD-L1) combined positive score (CPS) ≥10, *n* (%)	26 (41.9)	26 (40.6)	22 (36.1)
Microsatellite instability-high, *n* (%)	4 (6.5)	4 (6.3)	2 (3.3)
Adenocarcinoma of the stomach, *n* (%)	52 (83.9)	55 (85.9)	57 (93.4)
Number of metastases, *n* (%)			
0–2	39 (62.9)	42 (65.6)	44 (72.1)
≥3	21 (33.9)	20 (31.3)	16 (26.2)
Site of metastatic lesions, *n* (%)			
Adrenal glands	3 (4.8)	0	1 (1.6)
Bone	1 (1.6)	2 (3.1)	1 (1.6)
Liver	14 (22.6)	23 (35.9)	26 (42.6)
Lung	2 (3.2)	13 (20.3)	7 (11.5)
Lymph node	47 (75.8)	46 (71.9)	44 (72.1)
Peritoneum	37 (59.7)	29 (45.3)	28 (45.9)
Pleura	1 (1.6)	2 (3.1)	1 (1.6)
Other	10 (16.1)	7 (10.9)	2 (3.3)

^a^Sum of diameter of target lesions by RECIST v1.1.

### Efficacy—pembrolizumab monotherapy versus chemotherapy

**Figure 1:**
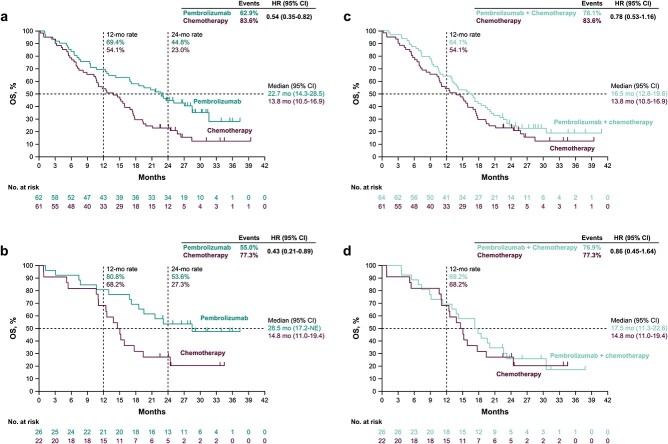
Kaplan–Meier estimates of overall survival for patients enrolled in Asia in the KEYNOTE-062 study. Pembrolizumab monotherapy versus chemotherapy in the (A) programmed death ligand 1 (PD-L1) combined positive score (CPS) ≥1 population and the (B) PD-L1 CPS ≥10 population. Pembrolizumab plus chemotherapy versus chemotherapy in the (C) PD-L1 CPS ≥1 population and the (D) PD-L1 CPS ≥10 population. HR, hazard ratio and NE, not estimable.

In the PD-L1 CPS ≥1 population, median OS was 22.7 months (95% CI, 14.3–28.5) with pembrolizumab versus 13.8 months (95% CI, 10.5–16.9) with chemotherapy (HR, 0.54; 95% CI, 0.35–0.82) (Fig. 1A). The 12-month and 24-month OS rates were 69.4% (95% CI, 56.3–79.2) and 44.8% (95% CI, 32.2–56.7) with pembrolizumab versus 54.1% (95% CI, 40.9–65.6) and 23.0% (95% CI, 13.4–34.1) with chemotherapy. In the PD-L1 CPS ≥10 population, median OS was 28.5 months (95% CI, 17.2–not estimable [NE]) with pembrolizumab versus 14.8 months (95% CI, 11.0–19.4) with chemotherapy (HR, 0.43; 95% CI, 0.21–0.89) (Fig. 1B). The 12-month and 24-month OS rates were 80.8% (95% CI, 59.8–91.5) and 53.6% (95% CI, 32.9–70.4) with pembrolizumab versus 68.2% (95% CI, 44.6–83.4) and 27.3% (95% CI, 11.1–46.4) with chemotherapy.

**Figure 2:**
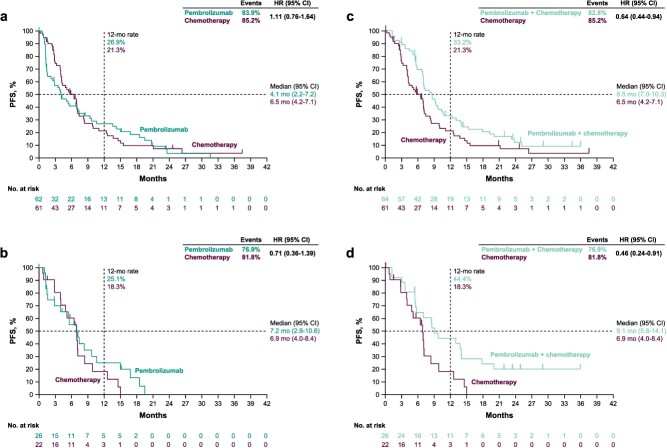
Kaplan–Meier estimates of progression-free survival for patients enrolled in Asia in the KEYNOTE-062 study. Pembrolizumab monotherapy versus chemotherapy in the (A) PD-L1 CPS ≥1 population and the (B) PD-L1 CPS ≥10 population. Pembrolizumab plus chemotherapy versus chemotherapy in the (C) PD-L1 CPS ≥1 population and the (D) PD-L1 CPS ≥10 population.

In the PD-L1 CPS ≥1 population, median PFS was 4.1 months (95% CI, 2.2–7.2) with pembrolizumab versus 6.5 months (95% CI, 4.2–7.1) with chemotherapy (HR, 1.11; 95% CI, 0.76–1.64) (Fig. 2A). In the PD-L1 CPS ≥10 population, median PFS was 7.2 months (95% CI, 2.9–10.6) with pembrolizumab versus 6.9 months (95% CI, 4.0–8.4) with chemotherapy (HR, 0.71; 95% CI, 0.36–1.39) (Fig. 2B).

In the PD-L1 CPS ≥1 population, 14 of 62 patients (22.6%) who received pembrolizumab versus 23 of 61 (37.7%) who received chemotherapy had an objective response, with complete responses in 4 of 62 patients (6.5%) versus 3 of 61 (4.9%), respectively (Table 2). Median DOR was 8.3 months (range, 1.4+ to 22.2+) with pembrolizumab versus 5.8 months (range, 2.7 to 23.6+) with chemotherapy (Supplementary Fig. 1A). In the PD-L1 CPS ≥10 population, 7 of 26 patients (26.9%) who received pembrolizumab versus 7 of 22 (31.8%) who received chemotherapy had an objective response, with complete responses in 2 of 26 patients (7.7%) versus 0 of 22 (0%), respectively (Table 2). Median DOR was 8.1 months (range, 1.4+ to 13.7) with pembrolizumab versus 5.3 months (range, 2.7–7.0) with chemotherapy (Supplementary Fig. 1B).

**Table 2:** Response summary for patients enrolled in Asia in the KEYNOTE-062 study by PD-L1 CPS cut-off

	PD-L1 CPS ≥1 (ITT)			PD-L1 CPS ≥10		
	Pembrolizumab *n* = 62	Pembrolizumab + Chemotherapy *n* = 64	Chemotherapy *n* = 61	Pembrolizumab *n* = 26	Pembrolizumab + Chemotherapy *n* = 26	Chemotherapy *n* = 22
Responses, *n*	14	34	23	7	16	7
Objective response rate, % (95% confidence interval)	22.6 (12.9–35.0)	53.1 (40.2–65.7)	37.7 (25.6–51.0)	26.9 (11.6–47.8)	61.5 (40.6–79.8)	31.8 (13.9–54.9)
Complete response, *n* (%)	4 (6.5)	7 (10.9)	3 (4.9)	2 (7.7)	4 (15.4)	0
Partial response, *n* (%)	10 (16.1)	27 (42.2)	20 (32.8)	5 (19.2)	12 (46.2)	7 (31.8)
Stable disease, *n* (%)	18 (29.0)	20 (31.3)	19 (31.1)	9 (34.6)	8 (30.8)	9 (40.9)
Progressive disease, *n* (%)	21 (33.9)	4 (6.3)	9 (14.8)	5 (19.2)	2 (7.7)	2 (9.1)
No assessment/nonevaluable, *n* (%)	9 (14.5)	6 (9.4)	10 (16.4)	5 (19.2)	0 (0.0)	4 (18.2)

### Efficacy—pembrolizumab plus chemotherapy versus chemotherapy

In the PD-L1 CPS ≥1 population, median OS was 16.5 months (95% CI, 12.8–19.6) with pembrolizumab plus chemotherapy versus 13.8 months (95% CI, 10.5–16.9) with chemotherapy (HR, 0.78; 95% CI, 0.53–1.16) (Fig. 1C). The 12-month and 24-month OS rates were 64.1% (95% CI, 51.0–74.5) and 24.6% (95% CI, 14.8–35.7) with pembrolizumab plus chemotherapy versus 54.1% (95% CI, 40.9–65.6) and 23.0% (95% CI, 13.4–34.1) with chemotherapy. In the PD-L1 CPS ≥10 population, median OS was 17.5 months (95% CI, 11.3–22.6) with pembrolizumab plus chemotherapy versus 14.8 months (95% CI, 11.0–19.4) with chemotherapy (HR, 0.86; 95% CI, 0.45–1.64) (Fig. 1D). The 12-month and 24-month OS rates were 69.2% (95% CI, 47.8–83.3) and 26.0% (95% CI, 11.0–43.8) with pembrolizumab plus chemotherapy versus 68.2% (95% CI, 44.6–83.4) and 27.3% (95% CI, 11.1–46.4) with chemotherapy.

In the PD-L1 CPS ≥1 population, median PFS was 8.5 months (95% CI, 7.0–10.3) with pembrolizumab plus chemotherapy versus 6.5 months (95% CI, 4.2–7.1) with chemotherapy (HR, 0.64; 95% CI, 0.44–0.94) (Fig. 2C). A total of 34 of 64 patients (53.1%) receiving pembrolizumab plus chemotherapy and 23 of 61 (37.7%) receiving chemotherapy had an objective response, with complete responses in 7 of 64 patients (10.9%) and 3 of 61 patients (4.9%), respectively (Table 2). Median DOR was 5.8 months (range, 1.4+ to 34.7+) with pembrolizumab plus chemotherapy versus 5.8 months (range, 2.7 to 23.6+) with chemotherapy (Supplementary Fig. 1C). In the PD-L1 CPS ≥10 population, median PFS was 9.1 months (95% CI, 5.6–14.1) with pembrolizumab plus chemotherapy versus 6.9 months (95% CI, 4.0–8.4) with chemotherapy (HR, 0.46; 95% CI, 0.24–0.91) (Fig. 2D). A total of 16 of 26 patients (61.5%) receiving pembrolizumab plus chemotherapy and 7 of 22 (31.8%) receiving chemotherapy had an objective response, with complete responses in 4 of 26 patients (15.4%) and 0 of 22 patients (0.0%), respectively (Table 2). Median DOR was 12.7 months (range, 3.8 to 34.7+) with pembrolizumab plus chemotherapy versus 5.3 months (range, 2.7–7.0) with chemotherapy (Supplementary Fig. 1D).

### Safety—all treatment arms

Treatment-related AEs occurred in 35 of 62 (56.5%), 62 of 62 (100%), and 53 of 57 patients (93.0%) receiving pembrolizumab, pembrolizumab plus chemotherapy and chemotherapy, respectively (Table 3 and Supplementary Table 2). Grade 3 or greater treatment-related AEs occurred in 12 of 62 (19.4%), 47 of 62 (75.8%) and 37 of 57 patients (64.9%), respectively. Treatment-related AEs resulted in discontinuation in 3 of 62 (4.8%), 16 of 62 (25.8%) and 12 of 57 patients (21.1%). One patient in the pembrolizumab plus chemotherapy therapy arm died of a treatment-related AE (malignant neoplasm progression). Immune-mediated AEs and infusion reactions occurred in 11 of 62 (17.7%), 14 of 62 (22.6%) and 4 of 57 patients (7.0%) with pembrolizumab, pembrolizumab plus chemotherapy and chemotherapy, respectively (Supplementary Table 3). Grade 3 or grade 4 immune-mediated AEs and infusion reactions occurred in 3 of 62 (4.8%), 4 of 62 (6.5%) and 0 of 57 patients (0%), respectively. Immune-mediated AEs and infusion reactions resulted in discontinuation in 1 patient in the pembrolizumab plus chemotherapy arm. No patient died of an immune-mediated AE or infusion reaction.

**Table 3:** Treatment-related adverse events^a^ for patients enrolled in Asia in the KEYNOTE-062 study

Treatment-related adverse event ≥ 5% in either group, *n* (%)	Pembrolizumab *n* = 62	Pembrolizumab + Chemotherapy *n* = 62	Chemotherapy *n* = 57
Pruritis	10 (16.1)	6 (9.7)	3 (5.3)
Decreased appetite	8 (12.9)	32 (51.6)	38 (66.7)
Rash	7 (11.3)	7 (11.3)	3 (5.3)
Diarrhoea	6 (9.7)	11 (17.7)	13 (22.8)
Fatigue	5 (8.1)	23 (37.1)	15 (26.3)
Rash maculo-papular	5 (8.1)	4 (6.5)	2 (3.5)
Anaemia	4 (6.5)	26 (41.9)	15 (26.3)
Aspartate aminotransferase increased	4 (6.5)	1 (1.6)	0
Hyponatremia	2 (3.2)	3 (4.8)	4 (7.0)
Nausea	2 (3.2)	34 (54.8)	31 (54.4)
Stomatitis	2 (3.2)	12 (19.4)	16 (28.1)
Vomiting	2 (3.2)	11 (17.7)	12 (21.1)
Blood creatinine increased	1 (1.6)	8 (12.9)	9 (15.8)
Constipation	1 (1.6)	6 (9.7)	11 (19.3)
Dizziness	1 (1.6)	5 (8.1)	3 (5.3)
Dry skin	1 (1.6)	3 (4.8)	3 (5.3)
Dysgeusia	1 (1.6)	6 (9.7)	5 (8.8)
Hypoalbuminemia	1 (1.6)	2 (3.2)	3 (5.3)
Malaise	1 (1.6)	8 (12.9)	5 (8.8)
Neutrophil count decreased	1 (1.6)	32 (51.6)	21 (36.8)
Peripheral oedema	1 (1.6)	4 (6.5)	1 (1.8)
Peripheral sensory neuropathy	1 (1.6)	13 (21.0)	6 (10.5)
Platelet count decreased	1 (1.6)	17 (27.4)	11 (19.3)
White blood cell count decreased	1 (1.6)	20 (32.3)	14 (24.6)
Alopecia	0	4 (6.5)	4 (7.0)
Asthenia	0	1 (1.6)	3 (5.3)
Dehydration	0	1 (1.6)	3 (5.3)
Hypomagnesaemia	0	5 (8.1)	6 (10.5)
Hypophosphataemia	0	2 (3.2)	3 (5.3)
Leukopenia	0	2 (3.2)	6 (10.5)
Mucosal inflammation	0	1 (1.6)	3 (5.3)
Neutropenia	0	10 (16.1)	11 (19.3)
Palmar-plantar erythrodysesthesia syndrome	0	25 (40.3)	21 (36.8)
Skin hyperpigmentation	0	4 (6.5)	2 (3.5)
Weight decreased	0	5 (8.1)	4 (7.0)

^a^Determined by the investigator to be related to the study drug.

## Discussion

In this subpopulation analysis of the phase 3 KEYNOTE-062 trial, first-line pembrolizumab monotherapy was associated with numerically improved OS compared with chemotherapy for patients enrolled in Asia with PD-L1–positive GC/GEJC. A greater benefit was observed with pembrolizumab monotherapy in this subgroup analysis compared with the overall KEYNOTE-062 study population, where OS with pembrolizumab monotherapy was found to be noninferior to chemotherapy in patients with PD-L1 CPS ≥1 but associated with longer OS in patients with PD-L1 CPS ≥10 (14). A trend for longer median OS with pembrolizumab plus chemotherapy in patients enrolled in Asia was observed compared to the overall study population, with no significant benefit observed versus chemotherapy (14). However, 12-month OS rates were consistently higher in pembrolizumab-treated patients enrolled in Asia compared with the overall population; this was particularly apparent for the PD-L1 CPS ≥10 population (12-month OS, 80.8%, 69.2% and 68.2% for pembrolizumab, pembrolizumab plus chemotherapy and chemotherapy, respectively) unlike the PD-L1 CPS ≥10 population in the overall study (12-month OS, 56.5%, 50.5% and 46.7%, respectively) (14). Assessment of the baseline characteristics revealed a higher proportion of patients in Asia with ECOG performance status 0 and a diagnosis of stomach cancer compared with the global KEYNOTE-062 population (14).

Longer survival times were expected in this analysis because Asian ethnicity, and residing in Asia, are favourable prognostic factors for patients with GC (15–18). In the randomized phase 3 CheckMate 649 study in patients with advanced GC/GEJC and oesophageal adenocarcinoma, first-line nivolumab plus chemotherapy versus chemotherapy alone demonstrated an OS benefit in the Asian subgroup regardless of PD-L1 status (all randomly assigned [*n* = 356]: HR, 0.76; 95% CI, 0.59–0.97; PD-L1 CPS ≥5 [*n* = 228]: HR, 0.64; 95% CI, 0.47–0.87) (19). Furthermore, in the randomized phase 2/3 ATTRACTION-4 study in Asian patients with advanced or recurrent GC/GEJC, first-line nivolumab plus chemotherapy versus chemotherapy significantly improved PFS but not OS (20). A recent meta-analysis comparing responses indicated that Asian patients may receive greater OS and PFS benefit with immune checkpoint inhibitor therapy compared with non-Asian patients (21). The present subgroup analysis of KEYNOTE-062 suggests that the proportion of patients enrolled in Asia with tumours that were MSI-H and PD-L1 CPS ≥1 (3.3%–6.5%) was comparable with the overall study population (5.5%–7.6%) (14). Furthermore, patients with GC/GEJC enrolled in Asia had a high**er** rate of transition to subsequent anticancer therapy compared with the overall study population in both the pembrolizumab monotherapy (83.9% vs 52.8%) and pembrolizumab plus chemotherapy (69.4% vs 47.2%) treatment arms (14), suggesting that they may be more likely to be treated with effective drugs. Additionally, factors beyond subsequent anticancer therapy may have contributed to the prolonged overall survival observed in the present subgroup analysis. Differences in overall survival between Western/global and Asian populations have been previously shown. In a subgroup analysis of the Trastuzumab for Gastric Cancer (ToGA) study, the lack of a survival benefit with trastuzumab plus chemotherapy versus chemotherapy in Japanese patients with advanced gastric or gastrooesophageal junction adenocarcinoma was attributed to imbalances in prognostic factors (e.g. prior gastrectomy, type of GC and number of metastatic lesions) between the treatment arms (22). These differences may have impacted clinical outcomes; however, the present analysis did not formally test for statistical significance between the study treatment arms because efficacy end points for the primary analysis of the KEYNOTE-062 study were not met.

PFS outcomes for patients enrolled in Asia were also similar to the overall study population for patients receiving chemotherapy, but the median PFS for patients receiving pembrolizumab plus chemotherapy was better for patients enrolled in Asia than the overall study population, especially amongst patients with PD-L1 CPS ≥10. Furthermore, no difference was apparent between treatment arms. However, more objective responses were achieved with pembrolizumab plus chemotherapy. Objective response rate amongst patients randomly assigned to receive pembrolizumab also appeared to be higher in the PD-L1 CPS ≥10 population, which is consistent with the overall population in KEYNOTE-062, as well as observations made in the KEYNOTE-061 study of second-line pembrolizumab monotherapy (14,23), highlighting the utility of high PD-L1 expression as a predictive biomarker to identify patients who will receive the greatest benefit from treatment with pembrolizumab (24,25). Patients with CPS ≥10, in particular, appeared to maintain response. However, PFS or ORR benefit was not correlated with OS benefit in the overall KEYNOTE-062 population, regardless of PD-L1 CPS (14).

The incidence of treatment-related AEs was also similar in this analysis compared to the overall study population analysis, with substantially lower rates observed in the pembrolizumab monotherapy arm. This finding suggests a treatment option for previously untreated patients who may be suitable candidates for chemotherapy; there is concern, however, regarding potential AEs, especially given that AEs occurring with chemotherapy may be more likely to occur in individuals from East Asian countries compared with individuals from Western countries (26). The high AE rates following treatment with chemotherapy (100% for pembrolizumab plus chemotherapy and 93.0% for chemotherapy alone) in this subpopulation analysis also reflect the high burden of toxic effects experienced by patients in Asia. Conversely, the substantially lower rates of AEs in patients treated with pembrolizumab monotherapy (56.5%) demonstrate that pembrolizumab offers a more favourable tolerability profile than chemotherapy when administered as a first-line treatment option for patients with GC/GEJC in Asia. Immune-mediated AEs in the overall KEYNOTE-062 population and the Asian subpopulation were also similar and consistent with reports from other phase 3 studies of pembrolizumab in GC/GEJC (11).

The role of pembrolizumab in the treatment of advanced GC remains to be determined; however, the current analysis adds to the existing body of evidence and is especially informative because GC is one of the most commonly diagnosed cancers and is a leading cause of death in Asia (3). Key limitations of this analysis include the lack of statistical comparisons; however, the results are largely consistent with the overall study population and the unblinded administration of pembrolizumab monotherapy, which may have influenced adherence and biased patient management.

Ongoing studies in the first-line treatment setting are evaluating pembrolizumab plus chemotherapy (KEYNOTE-859; NCT03675737) and pembrolizumab plus trastuzumab and chemotherapy (KEYNOTE-811; NCT03615326) and will provide additional support for the efficacy and safety of pembrolizumab in patients with GC/GEJC, including patients enrolled in Asia.

In conclusion, although statistical comparisons were not conducted, this subpopulation analysis of data for patients enrolled in Asia in the KEYNOTE-062 study indicates that pembrolizumab monotherapy was associated with numerically improved OS outcomes compared with chemotherapy alone for patients with advanced GC/GEJC with PD-L1 CPS ≥1 and CPS ≥10 tumours. Pembrolizumab monotherapy was also associated with a favourable tolerability profile compared with chemotherapy, which is consistent with observations from the overall KEYNOTE-062 study population.

## Abbreviations

BICR, blinded independent central review; CPS, combined positive score; CR, complete response; DOR, duration of response; EBV, Epstein–Barr virus; ECOG PS, Eastern Cooperative Oncology Group performance status; ERBB2, Erb-B2 receptor tyrosine kinase 2; GC, gastric cancer; GEJC, gastrooesophageal junction cancer; HER2, human epidermal growth factor receptor 2; ITT, intention to treat; MSI, microsatellite instability; MSI-H, high microsatellite instability; ORR, objective response rate; OS, overall survival; PD, progressive disease; PD-1, programmed death 1; PD-L1, programmed death ligand 1; PFS, progression-free survival; PR, partial response; Q3W, every 3 weeks; SD, stable disease; SOC, standard of care; TRAE, treatment-related adverse event

## Authors’ contribution

Conception, design or planning of the study: Yee Chao, Shi Rong Han, Shinichi Shiratori, Sukrut Shah and Kohei Shitara

Acquisition of data: Hironaga Satake, Keun-Wook Lee, Hyun Cheol Chung, Jeeyun Lee, Jen-Shi Chen, Takaki Yoshikawa, Kenji Amagai, Kun-Huei Yeh, Masahiro Goto, Yee Chao, Ka-On Lam and Kohei Shitara

Analysis of data: Kensei Yamaguchi, Ka-On Lam and Shi Rong Han

Interpretation of the results: Hironaga Satake, Keun-Wook Lee, Hyun Cheol Chung, Jen-Shi Chen, Takaki Yoshikawa, Yee Chao, Ka-On Lam, Sukrut Shah and Kohei Shitara

Drafting of the manuscript: Hironaga Satake and Kohei Shitara

Critically reviewing or revising of the manuscript for important intellectual content: Hironaga Satake, Keun-Wook Lee, Hyun Cheol Chung, Jeeyun Lee, Kensei Yamaguchi, Jen-Shi Chen, Takaki Yoshikawa, Kenji Amagai, Kun-Huei Yeh, Masahiro Goto, Yee Chao, Ka-On Lam, Shi Rong Han, Shinichi Shiratori, Sukrut Shah and Kohei Shitara

Final approval of the version to be published: All authors.

## Disclosures

Hironaga Satake reports research funding paid to his institution from Ono Pharmaceutical Co Ltd, Daiichi Sankyo and Taiho Pharmaceutical Co Ltd, and honoraria for lectures from Bristol Myers Squibb Co., Ltd., Bayer Co., Ltd., Chugai Pharmaceutical Co., Ltd, Daiichi Sankyo Co., Ltd., Eli Lilly Japan Co., Ltd., MSD Co., Ltd., Ono Pharmaceutical Co., Ltd., Sanofi Co., Ltd., Taiho Pharmaceutical Co., Ltd., Takeda Co., Ltd. and Yakult Honsha Co., Ltd.

Keun-Wook Lee reports grants paid to his institution for conducting clinical trials from Merck Sharp & Dohme LLC, a subsidiary of Merck & Co., Inc., Rahway, NJ, USA, AstraZeneca, Ono Pharmaceutical Co., Ltd., Merck KGaA, Pfizer, BeiGene, Astellas Pharma, ALX Oncology, Zymeworks, Macrogenics, Five Prime Therapeutics, Seagen, Bolt Therapeutics, Trishula Therapeutics, Oncologie, Pharmacyclics, LSK BioPharma, MedPacto, Green Cross Corp, ABLBIO, Y-BIOLOGICS, Genexine, Daiichi Sankyo, Taiho Pharmaceutical, InventisBio, Leap Therapeutics and Solasia; and personal fees from ISU ABXIS, Bayer, Daiichi Sankyo, Merck Sharp and Dohme, Bristol Myers Squibb, Vifor Pharma for consultation and Ono Pharmaceutical Co., Ltd., and Boryung for honoraria.

Hyun Cheol Chung reports consulting fees from Taiho, Celltrion, MSD, Eli Lilly, Bristol Myers Squibb, Merck Serono, Gloria, Beigene, Amgen and Zymework; honoraria from Merck-Serono and Eli Lilly; and that his institution received grants from Eli Lilly, GSK, MSD, Merck Serono, Bristol Myers Squibb, Ono Pharmaceutical Co., Ltd., Taiho, Amgen, Beigene, Incyte and Zymework.

Jeeyun Lee reports no conflicts of interest.

Kensei Yamaguchi reports receiving payment or honoraria from Taiho Pharm, Daiichi Sankyo, Eli Lilly Japan, Ono Pharmaceutical Co., Ltd., and Bristol Myers Squibb.

Jen-Shi Chen reports no conflicts of interest.

Takaki Yoshikawa reports lecture fees from Bristol Myers Squibb, MSD, Ono Pharmaceutical Co., Ltd., Taiho, Daiichi Sankyo, Lilly, Otsuka, Terumo, Covidien, EA Pharma, and AstraZeneca.

Kenji Amagai reports research funding from MSD, Taiho Pharmaceutical, Daiichi Sankyo, Nippon Zoki Pharmaceutical and Hisamitsu Pharmaceutical.

Kun-Huei Yeh reports no conflicts of interest.

Masahiro Goto reports receiving grants or contracts from Chugai Pharma, Nippon Kayaku and Taiho Pharmaceutical; and fees or honoraria for lectures, presentations, speakers bureaus, manuscript writing or educational events from Daiichi Sankyo Company, Limited, Ono Pharmaceutical Co., Ltd., Taiho Pharmaceutical, MSD K.K., Takeda Pharmaceutical Company Limited, Sumitomo Dainippon Pharma Co., Ltd., Yakult Pharmaceutical Industry Co., Ltd. and Eli Lilly Japan K.K.

Yee Chao reports no conflicts of interest.

Ka-On Lam reports receiving support from MSD; honoraria for lecturing and speaker bureaus from MSD, Bristol Myers Squib, Eli Lilly, Novartis, Bayer, Daiichi Sankyo, AstraZeneca, Merck, Amgen, Taiho, Sanofi; and serving as an advisory board member for MSD, Bristol Myers Squibb, Eli Lilly, Novartis, Bayer, Daiichi, Sankyo, AstraZeneca, Merck and Amgen.

Shi Rong Han is an employee of MSD K.K., Tokyo, Japan.

Shinichi Shiratori is an employee of and a stockholder in MSD K.K., Tokyo, Japan.

Sukrut Shah is an employee of Merck Sharp & Dohme LLC, a subsidiary of Merck & Co., Inc., Rahway, NJ, USA and has stock in Merck & Co., Inc., Rahway, NJ, USA.

Kohei Shitara reports employment/leadership position/advisory roles with Takeda, Pfizer, Ono Pharmaceutical Co., Ltd., MSD, Taiho Pharmaceutical, Novartis, AbbVie, GlaxoSmithKline, Daiichi Sankyo, Amgen, Boehringer Ingelheim; honoraria from Takeda and Bristol Myers Squibb; and research funding from Astellas, Ono Pharmaceutical Co., Ltd., Daiichi Sankyo, Taiho Pharmaceutical, Chugai, MSD, Medi Science, Eisai and Amgen.

## Data sharing

Merck Sharp & Dohme LLC, a subsidiary of Merck & Co., Inc., Rahway, NJ, USA (MSD) is obligated to protect the rights and privacy of trial participants. To fulfil the company’s obligation, MSD has a procedure in place for evaluating and fulfilling requests for sharing company clinical trial data with qualified external scientific researchers. As outlined on the MSD data sharing website (available at: http://engagezone.msd.com/ds_documentation.php), a detailed research proposal that includes the background and rationale, objectives of the research, a scientific hypothesis, statistical analysis plan and publication plan must be submitted through EngageZone along with the curricula vitae of all researchers, including the biostatistician. Completed applications will be promptly assessed for feasibility. If the request is considered to be feasible, a committee of MSD subject matter experts will assess the scientific validity of the request and the qualifications of the requestors. If the proposal is approved, the researcher must enter into a standard data-sharing agreement with MSD before anonymized data are provided; this is in line with data privacy legislation. There are circumstances that may prevent MSD from sharing the requested data, including country or region-specific regulations such as the European Union General Data Privacy Regulation. If the request is declined, it will be communicated to the investigator. MSD data-sharing metrics can be accessed at https://www.merck.com/clinical-trials/pdf/MicrositeDataSharingMetrics_20190711.pdf. 

## Funding

Funding for this study was provided by Merck Sharp & Dohme LLC, a subsidiary of Merck & Co., Inc., Rahway, NJ, USA. Grant number not applicable.

## Supplementary Material

Satake_et_al_FigS1_hyac188

Satake_et_al_Manuscript_Supplement_Highlighted_hyac188
