# Supplementary material for: Pembrolizumab or pembrolizumab plus chemotherapy versus standard of care chemotherapy in patients with advanced gastric or gastroesophageal junction adenocarcinoma: Asian subgroup analysis of KEYNOTE-062
Source: Jpn J Clin Oncol. 2022 Dec 18;53(3):221–9. doi: 10.1093/jjco/hyac188 (PMC9991501; doi:10.1093/jjco/hyac188)
Supplement: Satake_et_al_Manuscript_Supplement_Highlighted_hyac188 [file satake_et_al_manuscript_supplement_highlighted_hyac188.doc]

**SUPPLEMENT to** *Japanese Journal of Clinical Oncology*

Pembrolizumab or pembrolizumab plus chemotherapy versus standard-of-care chemotherapy in patients with advanced gastric or gastroesophageal junction adenocarcinoma: Asian subgroup analysis of KEYNOTE-062

Hironaga Satake, Keun-Wook Lee, Hyun Cheol Chung, Jeeyun Lee, Kensei Yamaguchi, Jen-Shi Chen, Takaki Yoshikawa, Kenji Amagai, Kun-Huei Yeh, Masahiro Goto, Yee Chao, Ka-On Lam, Shi Rong Han, Shinichi Shiratori, Sukrut Shah, Kohei Shitara

**Corresponding author**: Hironaga Satake

Department of Medical Oncology, Kochi Medical School

Email: satakeh@kochi-u.ac.jp

**Supplementary Table 1.** Anticancer therapy following discontinuation in patients enrolled in Asia in the KEYNOTE-062 study

|  | **Pembrolizumab *n* = 62** | **Pembrolizumab + Chemotherapy**  ***n* = 62** | **Chemotherapy**  ***n* = 57** |
| --- | --- | --- | --- |
| Received any subsequent therapy | 52 (83.9) | 43 (69.4) | 42 (73.7) |
| Antineoplastic and immunomodulating agents (≥10% in either treatment arm) | | | |
| Oxaliplatin | 38 (61.3) | 7 (11.3) | 4 (7.0) |
| S-1 | 33 (53.2) | 7 (11.3) | 4 (7.0) |
| Paclitaxel | 32 (51.6) | 36 (58.1) | 33 (57.9) |
| Ramucirumab | 22 (35.5) | 28 (45.2) | 26 (45.6) |
| Irinotecan hydrochloride | 18 (29.0) | 15 (24.2) | 14 (24.6) |
| Fluorouracil | 14 (22.6) | 5 (8.1) | 4 (7.0) |
| Capecitabine | 12 (19.4) | 2 (3.2) | 2 (3.5) |
| Cisplatin | 8 (12.9) | 0 | 0 |
| Nivolumab | 6 (9.7) | 7 (11.3) | 16 (28.1) |

Data are *n* (%).

**Supplementary Table 2**. Adverse event summary for patients enrolled in Asia in the KEYNOTE-062 study

| ***n* (%)** | **Pembrolizumab *n* = 62** | **Pembrolizumab + Chemotherapy**  ***n* = 62** | **Chemotherapy**  ***n* = 57** |
| --- | --- | --- | --- |
| Any AE | 59 (95.2) | 62 (100) | 57 (100) |
| Grade 3–5 | 22 (35.5) | 52 (83.96) | 45 (78.9) |
| Serious | 15 (24.2) | 19 (30.6) | 27 (47.4) |
| Led to discontinuation | 6 (9.7) | 18 (29.0) | 15 (26.3) |
| Led to death | 2 (3.2) | 1 (1.6) | 2 (3.5) |
| Any treatment-related AE^a^ | 35 (56.5) | 62 (100) | 53 (93.0) |
| Grade 3–5 | 12 (19.4) | 47 (75.8) | 37 (64.9) |
| Serious | 8 (12.9) | 13 (21.0) | 12 (21.1) |
| Led to discontinuation | 3 (4.8) | 16 (25.8) | 12 (21.1) |
| Led to death^b^ | 0 | 1 (1.6) | 0 |

*AE,* adverse event.

^a^Determined by the investigator to be related to the study drug.

^b^Deaths attributed to study treatment occurred in 1 (1.6%) of 62 patients in the pembrolizumab plus chemotherapy arm (malignant neoplasm progression).

**Supplementary Table 3.** Immune-mediated adverse events^a^ and infusion reactions for patients enrolled in Asia in the KEYNOTE-062 study

| ***n* (%)** | **Pembrolizumab *n* = 62** | **Pembrolizumab + Chemotherapy**  ***n* = 62** | **Chemotherapy**  ***n* = 57** |
| --- | --- | --- | --- |
| Any immune-mediated AE or infusion reaction | 11 (17.7) | 14 (22.6) | 4 (7.0) |
| Grade 3 or grade 4 | 3 (4.8) | 4 (6.5) | 0 |
| Led to discontinuation | 0 | 1 (1.6) | 0 |
| Led to death | 0 | 0 | 0 |
| All immune-mediated AEs or infusion reactions | | | |
| Colitis | 2 (3.2) | 2 (3.2) | 1 (1.8) |
| Hypothyroidism | 2 (3.2) | 5 (8.1) | 3 (5.3) |
| Pneumonitis | 2 (3.2) | 2 (3.2) | 0 |
| Adrenal insufficiency | 1 (1.6) | 2 (3.2) | 0 |
| Hyperthyroidism | 1 (1.6) | 0 | 0 |
| Hypophysitis | 1 (1.6) | 0 | 0 |
| Myositis | 1 (1.6) | 0 | 0 |
| Nephritis | 1 (1.6) | 0 | 0 |
| Thyroiditis | 1 (1.6) | 0 | 0 |
| Type 1 diabetes mellitus | 1 (1.6) | 0 | 0 |
| Infusion reaction | 0 | 1 (1.6) | 0 |
| Severe skin reactions | 0 | 2 (3.2) | 0 |
| Uveitis | 0 | 1 (1.6) | 0 |

*AE,* adverse event.

^a^Defined for the safety profile of pembrolizumab as events with potentially treatment-related immunologic causes.

**Supplementary Figure 1.** Kaplan-Meier estimates of DOR for patients enrolled in Asia in the KEYNOTE-062 study. Pembrolizumab monotherapy versus chemotherapy in the (A) PD-L1 CPS ≥1 population and the (B) PD-L1 CPS ≥10 population. Pembrolizumab plus chemotherapy versus chemotherapy in the (C) PD-L1 CPS ≥1 population and the (D) PD-L1 CPS ≥10 population. *CPS,* combined positive score; *HR,* hazard ratio; *PD-L1,* programmed death ligand 1; *SOC,* standard of care.
